# Prevalence of Undiagnosed Hypertension in the Emergency Department

**DOI:** 10.5812/traumamon.7328

**Published:** 2014-01-25

**Authors:** Ali Arhami Dolatabadi, Maryam Motamedi, Hamidreza Hatamabadi, Hossein Alimohammadi

**Affiliations:** 1 Department of Emergency Medicine, Imam Hossein Medical Center, Shahid Beheshti University of Medical Sciences, Tehran, IR Iran; 2 Safety Promotion and Injury Prevention Research Center, Shahid Beheshti University of Medical Sciences, Tehran, IR Iran

**Keywords:** Hypertension, Emergency Department, Hospital, Blood Pressure, prevalence

## Abstract

**Background:**

Hypertension (HTN) is a serious health problem that threatens one fourth of the adult population in some countries.

**Objectives:**

This study aimed to assess the prevalence and outcome of undiagnosed hypertensive patients admitted to the emergency department.

**Materials and Methods:**

This cross-sectional study was conducted from March 2009 to March 2010 at Imam Hossein Medical and Educational Center, Teheran, Iran. A total of 2070 patients aged 18 years and older were admitted to the emergency department without previous HTN history. Blood pressure was taken and repeated 10 minutes later if initial systolic blood pressure ≥ 140 mmHg or diastolic blood pressure ≥ 90 mmHg. Those who matched the inclusion criteria entered the study for further follow-up. A numerical pain score was also used for pain intensity assessment. Chi-Square and Mann Whitney U tests were performed to compare differences between sex, age and education of the participants.

**Results:**

Based on the inclusion criteria, 346 patients entered the study, out of which 168 qualified for further evaluation and follow-up. Forty eight patients (28.6%) were finally diagnosed with high blood pressure. Our study showed that the prevalence of undiagnosed HTN was 4.8%. Significant differences between blood pressure, age, pain score and education level (P < 0.001) were found. This implies that old age, poor education and low pain score are positively associated with hypertension.

**Conclusions:**

Blood pressure readings in emergency departments should not be readily attributed to pain or anxiety. Diagnosis must be based on meticulous follow-up and precise examinations.

## 1. Background

Hypertension (HTN) is a serious health hazard. In some countries, nearly one fourth of the adult population suffers from HTN (1). About 75% of patients with chronic blood pressure (BP) are aware of having HTN, among whom one fourth to one half get proper treatment (2). Proper management of HTN can substantially reduce stroke risk and mortality rate (2). Many studies have shown that most of the patients with high blood pressure readings in the emergency department have no history of HTN and, in fact, suffer from a chronic undiagnosed HTN (3, 4). Emergency department personnel usually ascribe high BP to pain and anxiety. This causes the underlying HTN to stay unrecognized (1). 

## 2. Objectives

In this prospective cross-sectional study the aim was to follow up the patients with no history of HTN but with high BP readings to further assess the prevalence of undiagnosed HTN. 

## 3. Materials and Methods

A prospective cross-sectional study was designed to assess the prevalence of undiagnosed HTN among the patients admitted to the Emergency Department of the Imam Hossein Medical and Educational Center, Teheran, Iran, from March 2009 to March 2010. This study was approved by the Ethics Committee of Shahid Beheshti University of Medical Sciences, Teheran, Iran. All trauma and non-trauma patients with elevated BP were admitted to the triage room first and then referred to the emergency physician. According to the 7th Report of the Joint National Committee on Prevention, Detection, and Treatment of High Blood Pressure, HTN is defined as diastolic BP ≥ 90 mmHg or systolic BP ≥ 140 mmHg (5). Patients over 18 years-old with elevated BP and no history of HTN were held under observation for 10 minutes. If the BP remained high at the second reading, they were enrolled in the study. Pregnant women and patients with an arm circumference greater than 45 cm or lower than 19 cm were excluded. A structured questionnaire was used for each patient in order to collect contact information, demographic data, medical history, reason for admission and a complete list of medications. Pain intensity was recorded using a numerical pain score with two anchor points from 0 to 10 (0 indicating no pain and 10 indicating the worst pain ever experienced), just before the second BP measurement. If the patient did not need urgent medical help for high BP, he/she was discharged and advised for further follow-up one month later. In case the patient was found to have a high BP reading during the follow up visit, he was then referred to the internist for medical treatment. Blood pressure was measured with a standardized mercury sphygmomanometer with the patient in supine position with the right arm at the level of the heart and after 5 minutes of rest. Statistical analysis was carried out using the SPSS software, version 17 (IBM Corp., Armonk, New York, USA). The Chi-Square and Mann Whitney U tests were used for prevalence comparison of HTN between sex, education and age groups ( P < 0.05 was considered statistically significant).

## 4. Results

Two thousand and seventy patients (65.9% men) underwent BP measurement, out of which 346 (16.7%) had two elevated BP readings at 10 minutes intervals and entered the study. The sample had a mean (± SD) age 46.7 ± 12.4 years. The demographic data is summarized in Table 1. Only 168 out of 346 patients (48.5%) returned for follow-up. In the follow up visit, 120 patients (71.4%) had normal BP and 48 (28.6%) had high blood pressure (58.3% males) (Table 2). Since samples were randomly referred, we can infer that 99 cases out of 346 would have statistically been diagnosed as hypertensive, if they all had taken part in the follow up. Therefore, the undiagnosed hypertension prevalence in this study seemed to be about 4.8%. The mean systolic and diastolic BP in the HTN patients was 145.8 ± 48.6 mmHg and 83.5 ± 17.2 mmHg, respectively. Ten (20.8%) out of 48 patients with newly diagnosed HTN came to the ED for trauma and the rest 38 (79.2%) were admitted for non-traumatic reasons. There was no correlation between newly diagnosed HTN and sex (P = 0.12). On the contrary, a statistically significant relation between age and HTN was found (P = 0.001). In newly diagnosed hypertensive patients, 13 (27%) had a positive family history of HTN. There was no significant difference between familial history and BP (P = 0.55). Twenty six (54%) of the newly diagnosed HTN patients and 101 (84%) of the non-hypertensive ones had pain scores greater than one during their initial emergency stay. We found a statistically significant relation between the pain score and newly diagnosed hypertensive cases (P < 0.0001). Higher pain scores upon admission were less common among diagnosed hypertensive patients. There was a statistically significant relation between education and HTN diagnosis (P < 0.0001). A statistically significant relation was also found between emergency admission causes and HTN diagnosis, with non-traumatic patients showing higher HTN diagnosis at follow-up (P < 0.001). 

**Table 1.** Sex Frequency

	Frequency	Percent	Cumulative Percent
**Male**	111	66.1	66.1
**Female**	57	33.9	100
**Total**	168	100	

**Table 2.** Hypertension Frequency at Follow Up Visit

	Frequency	Percent	95% Confidence Interval
**No hypertension**	120	71.4	[63.1 - 78.4]
**Hypertension**	48	28.6	[21.0 - 35.3]

## 5. Discussion

In this study, one month follow-up depicted a prevalence of 4.8% newly diagnosed HTN, which was positively associated with low education, old age and non-traumatic cases. Epidemiological studies have reported different prevalence of undiagnosed HTN in apparently healthy individuals (5). For example, Fleming et al. (2004) reported 5% HTN cases during ED admission that would be diagnosed as sustained HTN in follow-up assessment (6). Svenson et al. (2006) also demonstrated that 8% of patients referred to the ED suffered from unrecognized HTN (1). Furthermore, in the study of Tilman et al. (2004), the prevalence of asymptomatic HTN in patients admitted to the ED was significantly higher (16%) than in other studies (7). Since the ED is the only means of communication with the clinic in some cases, Tilman et al. concluded that hypertension screening in the ED would be the best opportunity for patient evaluation (7). This will promote management and reduce morbidity and mortality related to HTN (8). Also, we found a positive association between pain score on admission and HTN. In our study, high pain scores on admission were less common among diagnosed hypertensive patients during the follow-up assessment. However, a high pain score can actually cause transient high BP in some cases. Backer et al. showed that pain tolerance among healthy and hypertensive patients in the ED was not significantly different from the pain score during the follow-up period (9). Tanabe et al. also reported a positive association between pain score and HTN, but they found no link between the anxiety level and HTN (8). Other studies have shown the same irrelevant findings between pain score and high BP (1, 10, 11). Since any value on a pain scale is subjective, ED administered or self-administered pain killers, together with previous history of chronic pain, can affect or influence pain interpretation (12). We found a statistically significant relation between education and undiagnosed HTN. This can be attributed to the fact that less educated individuals pay less attention to their health status and are more prone to have an unrecognized medical condition like HTN for long periods of time. It also indicates the importance of the ED as a suitable place to detect unrecognized HTN in less educated people who are the regular ED attendees, even if they do not need emergency care, instead of using other means for seeking health assistance. One limitation of the present study accounts for the high percentage of subjects who did not refer again for the follow up assessment. This might somehow imply that these findings cannot represent the whole population. Those who came for the follow up may well be those who were more concerned about their health. Therefore, we might have lost some undiagnosed hypertensive cases and which might go beyond the actual figure. Another limitation of the present study is that the pain level was not measured during the second visit. It would have been possible to compare pain levels in pre and post follow up visits, if they were measured. Conceivably, a better interpretation and conclusion of the effect of pain on HTN could be made. This study is the first HTN monitoring and assessment among patients referred to the EDs of the Iranian and Persian Gulf region hospitals to determine the prevalence of HTN. The selected hospital for this study is considered among the most populated referral hospitals from different regions of the country and the findings can be applied on a national scale. Our findings show that a striking percentage of individuals referred to the ED may suffer from unrecognized hypertension. Therefore, we should not consider these findings as transient and unimportant and instead, should pay particular attention and promote careful follow-up. Accordingly, EDs should be considered as appropriate places to screen for HTN, especially among low income patients who have limited access to healthcare services. 
